# Use of chemostat cultures mimicking different phases of wine fermentations as a tool for quantitative physiological analysis

**DOI:** 10.1186/1475-2859-13-85

**Published:** 2014-06-13

**Authors:** Felícitas Vázquez-Lima, Paulina Silva, Antonio Barreiro, Rubén Martínez-Moreno, Pilar Morales, Manuel Quirós, Ramón González, Joan Albiol, Pau Ferrer

**Affiliations:** 1 Departament d’Enginyeria Química. Escola d’ Enginyeria, Universitat Autònoma de Barcelona, Bellaterra, Cerdanyola del Vallès, Spain; 2 Instituto de Ciencias de la Vid y del Vino, CSIC, Universidad de La Rioja, Gobierno de La Rioja, Logroño, Spain; 3 Present affiliation: Escuela de Ingeniería Bioquímica, Universidad Católica de Valparaíso, Valparaíso, Chile; 4 Present affiliation: Bioingenium s.l, Barcelona, Catalonia, Spain; 5 Present affiliation: Greenaltech s.l, Barcelona, Catalonia, Spain; 6 Present affiliations: Quercus Europe s.l., L’Hospitalet de Llobregat, Catalonia, Spain; 7 Present affiliations: Universidad Internacional de la Rioja, Logroño, La Rioja, Spain; 8 Present affiliation: Evolva Biotech A/S, Copenhagen, Denmark

## Abstract

**Background:**

*Saccharomyces cerevisiae* is the most relevant yeast species conducting the alcoholic fermentation that takes place during winemaking. Although the physiology of this model organism has been extensively studied, systematic quantitative physiology studies of this yeast under winemaking conditions are still scarce, thus limiting the understanding of fermentative metabolism of wine yeast strains and the systematic description, modelling and prediction of fermentation processes. In this study, we implemented and validated the use of chemostat cultures as a tool to simulate different stages of a standard wine fermentation, thereby allowing to implement metabolic flux analyses describing the sequence of metabolic states of *S. cerevisae* along the wine fermentation.

**Results:**

Chemostat cultures mimicking the different stages of standard wine fermentations of *S. cerevisiae* EC1118 were performed using a synthetic must and strict anaerobic conditions. The simulated stages corresponded to the onset of the exponential growth phase, late exponential growth phase and cells just entering stationary phase, at dilution rates of 0.27, 0.04, 0.007 h^−1^, respectively. Notably, measured substrate uptake and product formation rates at each steady state condition were generally within the range of corresponding conversion rates estimated during the different batch fermentation stages.

Moreover, chemostat data were further used for metabolic flux analysis, where biomass composition data for each condition was considered in the stoichiometric model. Metabolic flux distributions were coherent with previous analyses based on batch cultivations data and the pseudo-steady state assumption.

**Conclusions:**

Steady state conditions obtained in chemostat cultures reflect the environmental conditions and physiological states of *S. cerevisiae* corresponding to the different growth stages of a typical batch wine fermentation, thereby showing the potential of this experimental approach to systematically study the effect of environmental relevant factors such as temperature, sugar concentration, C/N ratio or (micro) oxygenation on the fermentative metabolism of wine yeast strains.

## Background

During the last decades concern on climate change has increased and it is nowadays well recognised as one of the most important environmental problems faced on Earth. Climate change is already having significant impacts on the world’s physicochemical, biological and human systems. Agriculture is one of the main sectors affected by this phenomenon [1-3], with viticulture and viniculture being no exception [4-6]. Climate change alters crop yields and grape quality, and variations in anthocyanin, malic acid or sugar content could ultimately affect wine quality [7]. This is particularly important in regions of countries such as Spain, France, United States, Chile or Australia, where wine has developed as a key economic sector with broad historical, social, and cultural identity derived from grape growing and production.

Besides, although many of the wine properties and production methods are grape-related, there are numerous features that are dependent on the yeast strain used. These include fermentation performance (e.g. tolerance to stress and the ability to efficiently utilise carbon and nitrogen sources), downstream wine processing (e.g. improved protein and polysaccharide clarification, cell flocculation and sedimentation properties), modulation of alcohol content, levels of both desirable (e.g. resveratrol) and undesirable (e.g. ethyl carbamate) chemical compounds, as well as the modulation of the organoleptic properties resulting from the hundreds of metabolites and flavour compounds that are either produced or liberated from precursors in the grape juice during wine fermentation, including esters, higher alcohols, volatile acids, phenols and thiols [8].

For these reasons, wine producers have launched different initiatives over the last years that aim on one side, to understand and mitigate the impacts of global warming on winemaking and, on the other, to improve their knowledge base on yeast physiology under winemaking conditions for the optimisation of production processes.

In the past recent years, systems biology tools have been extensively used to study the physiology of the model yeast *S. cerevisiae*. Nevertheless, the application of such tools to the understanding of yeast physiology under winemaking conditions is still limited [9-14].

Wine fermentations are operated in batch mode. Due to the intrinsic nature of this process, yeast metabolism undergoes a series of adaptive changes in response to the initial stressing conditions as well as to the continuous environmental variations that take place. Specifically, yeasts have to adapt to the characteristics of the grape must, that is, high osmolarity due to the high sugar concentration, low pH and presence of sulphites. Also, heat is generated and ethanol is accumulated to high concentrations (ca. 12 ~ 15%) as a result of the fermentative activity of yeast. Moreover, limited availability of assimilable nitrogen sources and oxygen leads to rapid nutrient deficiency after a first growth phase. Notably, most of the ethanol is produced during the later fermentation stages, i.e. at near zero growth rates and stationary phase.

Systematic profiling of yeast metabolism under winemaking conditions is required in order to understand and predict the effect of environmental changes and distinct genetic backgrounds on wine fermentations, as well as to perform a rational optimisation of fermentation processes. Among the strategies for systems-level analysis of cell metabolism, Metabolic Flux Analysis (MFA) has been extensively applied in many physiological studies of yeast, for example to quantify the impact of growth conditions or genetic modifications on metabolic pathway activities [15-24].

Attaining metabolic steady states is crucial to investigate metabolic pathways using MFA methodologies, including MFA based on ^13^C isotopic labelling. Moreover, characterisation of cellular metabolism using isotopic tracers such as ^13^C-labelled substrates is much more convenient in chemically defined media with one or two carbon sources at a relatively low concentration. Nevertheless, laboratory scale fermentations mimicking wine fermentations may be more complicated due to the higher complexity associated with the medium (composed of several carbon sources including glucose, fructose and amino acids), as well as co-consumption and secretion of substrates and metabolites. To date, several experimental approaches have been proposed for MFA studies of yeast growing under wine making conditions. Varela and co-workers [20] reported one of the first examples of the use of MFA to characterise the distribution of carbon fluxes in a wine yeast strain under winemaking conditions. In this study, changes in the wine fermentation process are assumed to be slow in comparison to intracellular metabolite dynamics, that is, intracellular metabolite pools were assumed to be at pseudo-steady state in a given fermentation point. Therefore, metabolic fluxes can be estimated from the measurements of substrate uptake and product formation rates at a given fermentation stage. Recently, this basic approach has been further extended using a genome-scale metabolic model [13]. Also, dynamic metabolic flux balance approaches, linking process variables and metabolic fluxes, have been proposed [25,26], allowing for the simulation and prediction of winemaking fermentation kinetic profiles.

Alternatively, chemostat cultures have been extensively used for quantitative physiology analyses of yeast cells. Nevertheless, their application to the study of wine fermentations has been so far very limited. For instance, this approach has been used to investigate the effect of growth parameters such as temperature [9] or dissolved oxygen concentration [14] on yeast cells. However, these studies were carried out under environmental conditions not strictly resembling wine fermentations (for instance, they used glucose as a single carbon source). Moreover, conventional continuous cultures were never applied to the study of the different growth stages of a typical wine fermentation. Interestingly, recent studies by Clement and co-workers [27,28] using a continuous multistage bioreactor connecting two or more tanks in series, shows the potential of chemostat cultures for reproducing the different stages of a batch process. However, no MFA studies have been reported on such kind of complex experimental set-up.

In this study, we propose the utilization of classic chemostat cultures to obtain metabolic steady states mimicking different physiological states found in different time points (stages) along a classical wine-making fermentation process, operated in batch mode. This has allowed for the quantitative physiological analysis of each fermentation phase, as exemplified by the MFA performed with the obtained datasets.

## Results

### Batch fermentation process

**Figure 1:**
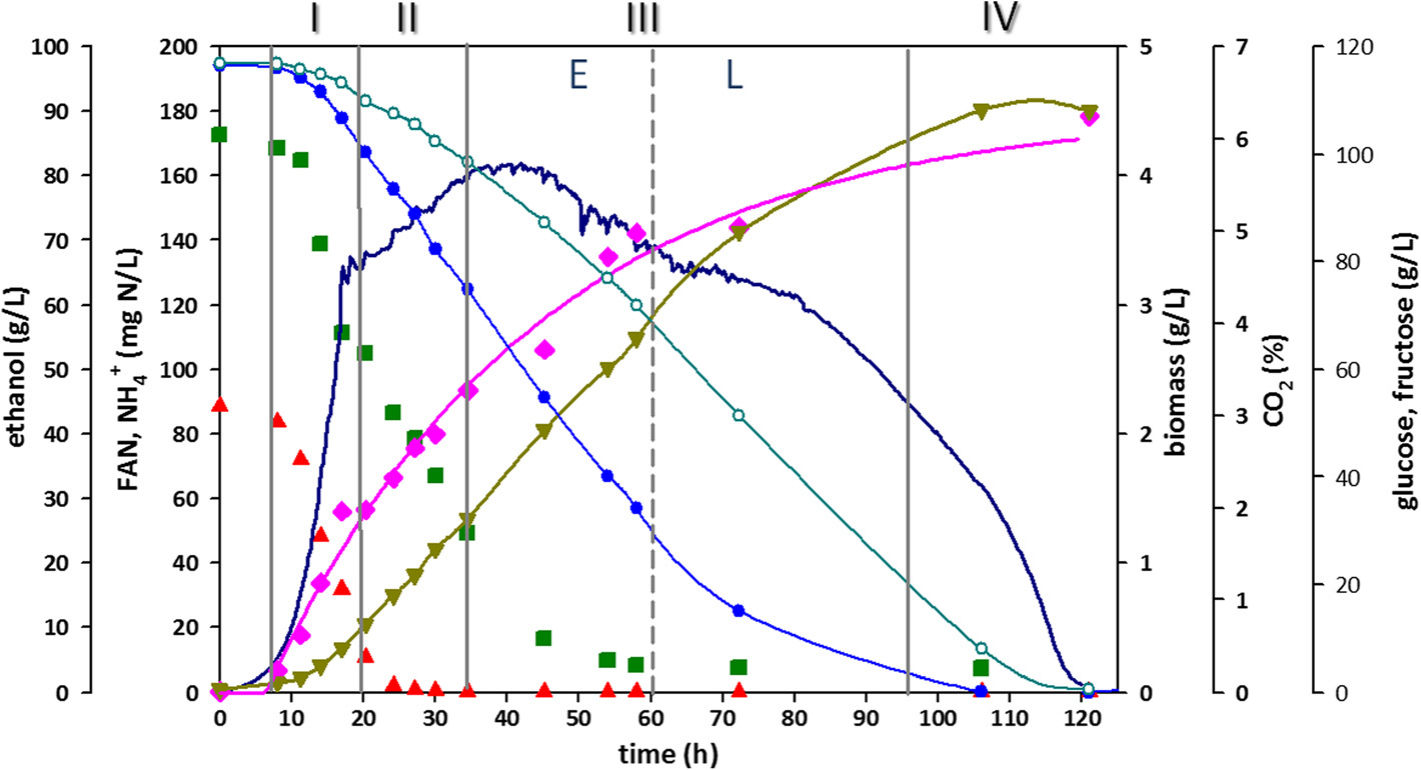
**Batch fermentation profile.** Vertical continuous grey lines indicate the proposed phases. Discontinued grey line divides phase III into early (E) and late (L) subphases. (Red triangle) $NH_{4}^{+}$, (Square olive green) FAN, **−** CO_2_, (Diamond pink) biomass, (Inverted triangle olive green) ethanol, (Blue circle) glucose, (Green circle) fructose.

Initially, the fermentation profile for strain EC1118 was studied in a standard batch fermentation at 28°C under strict anaerobic conditions with a synthetic must mimicking a typical natural must (i.e. 240 g/L sugars, with equimolar amounts of glucose and fructose, 200 mg/L yeast assimilable nitrogen (YAN), pH 3.5, and sulphites) [29], as shown in Figure 1. As it can be observed, fermentation finished after 110 ~ 120 h, when carbon sources were completely depleted and CO_2_ concentration in the exhaust gas was virtually zero. Fermentation evolution shows that glucose was the preferred carbon source since it was consumed and depleted quicker than fructose, following the behaviour already described by other authors [10,20,30,31]. Ethanol was synthesised throughout the process reaching a maximum concentration close to 12% (v/v) at around 106 h, when production stopped because carbon sources became almost depleted. In the case of CO_2_ production, it evolved exponentially during the initial stage of the fermentation, followed by a period when started to slow down; this period was coincident with a substantial reduction of the nitrogen content of the medium. Biomass grew exponentially during the early stages of the process (8–17 h); after that first phase, growth started to slow down as a result of a substantial reduction of the nitrogen content (both in the form of NH_4_^+^ and as free α-amino acids (FAN)) of the medium, as shown in Figure 1; at later stages of the process (after 60 h), a stationary growth phase was gradually reached due to the limitation of nitrogen sources and increasing ethanol concentration of the medium. Glycerol, the most abundant product of yeast fermentation after ethanol, was also synthesised during the process, reaching a final concentration of 10 g/L, which is within the range of concentrations commonly found in these processes [32]. Other products such as acetic, succinic and lactic acid were also produced with final concentrations of 0.7, 0.8 and 0.3 g/L, respectively. These results were consistent to those previously reported [20] for similar processes. Moreover, profiles determined in this study were also comparable to those described for industrial process (data not shown), thus indicating that our laboratory set-up adequately mimics the standard wine fermentations and the results could then be used as the basis for the modelling and simulation processes.

**Figure 2:**
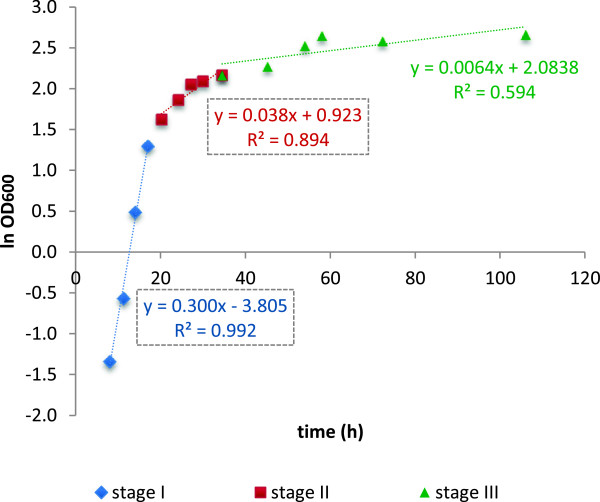
**Estimation of the specific growth rates (μ).** Calculated growth rates for the three first culture stages of the batch fermentation process. (Blue diamond) represents the first stage where μ_max_ is achieved, (Red Sqaure) represents the second or transition stage, and (Green triangle) represents the stage III. The slope of the regression equations is equivalent to the calculated μ.

Next step was defining and choosing the phases from the batch process to be reproduced as a series of steady states in continuous cultures. As a first approach, the specific growth rates observed during the batch process were the criteria used for the selection of the two first stages to be simulated, corresponding to growing cells. In particular, Phase I corresponded to mid-exponential growth phase and Phase II to late exponential growth phase. Figure 2 shows an example of how these two phases were defined; in this figure, the first curve represents the interval where the maximum specific growth rate (no nutrient limitations) is achieved and ethanol is produced at a maximum rate, and the second one corresponds to a transition growth phase where CO_2_ and ethanol production slow down, $NH_{4}^{+}$ is depleted and growth is sustained solely on free amino nitrogen. For the first phase (8–17 h), a μ_max_ = 0.29 h^−1^ ± 0.01 was calculated as an average from four different batch replicates, while for the transition period –or late exponential growth phase- (20–35 h), a μ_trans_ = 0.04 h^−1^ ± 0.01 was obtained. At the end of the transition phase the CO_2_ production rate (CER) peaked, gradually declining thereafter along the following fermentation stage (stationary phase).

Phase III corresponded to the onset of the stationary phase. During this period (35–106 h) growth rate gradually declined from 0.04 to 0 h^−1^, along with the decline in CO_2_ production and accumulation of ethanol. An average μ of 0.007 h^−1^ was estimated for this phase (Figure 2). At the end of this phase, sugar substrates were virtually exhausted and most of the amino acids consumed. At Phase IV (stationary phase, corresponding to the 106–120 h period), the cells were no longer proliferating (or cell proliferation was equal to cell death), the remaining sugar (fructose) was completely depleted, CO_2_ production abruptly fell down and ethanol reached its maximum (11.7%). This value is slightly lower to that obtained in similar studies [20] and large scale winemaking fermentations with similar initial sugar content (data not shown). This was probably due to limited ethanol stripping as a result of reactor mixing and nitrogen gas sparging. Indeed, this was estimated to be about 0.17 g ethanol/L · h in the Phase I (A. Barreiro, unpublished results).

**Figure 3:**
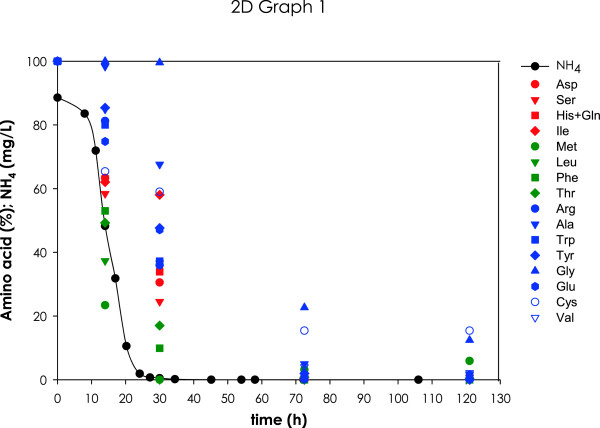
**Amino acid consumption profiles.** Consumption of ammonium and amino acids during batch fermentation of strain EC1118. The residual $NH_{4}^{+}$ concentration is shown by black solid circles and black line. Early consumption amino acids (Met, Leu, Phe, Thr) are shown in green symbols. Intermediate consumption amino acids (Asp, Ser, His, Gln, Ile) are shown in red symbols. Late consumption amino acids (Arg, Ala, Trp, Tyr, Gly, Glu, Cys, Val) are shown in blue symbols. The residual concentrations of $NH_{4}^{+}$ and amino acids are expressed as percentage of the initial concentration.

With the exception of proline, which is not assimilated in absence of oxygen [33], all the nitrogen sources were utilized. The amino acids and ammonium were assimilated with variable kinetic patterns, following an order of use coherent with those reported in similar recent studies [34] (Figure 3). In particular, amino acids such as Arg, Ala, Trp, Tyr and Gly were consumed at the later stages of the growth phase, after ammonium and some other nitrogen sources had been exhausted, while the rest of amino acids were assimilated earlier, simultaneously to ammonium, or even before (e.g. Leu and Met). Co-consumption of ammonium and amino acids has been previously described by [35,36].

Cell viability, cell size and accumulation of reactive oxygen species (ROS) were monitored along the fermentation by flow cytometry. Consistent with previous studies [37,38], there was a significant ROS accumulation (% of DHE-stained cells) during fermentation, reaching over 70% of the cell population during the early growth phases (Phases I and II). However, these values decreased progressively to about 30% during Phase III, suggesting an adaptation to increased ethanol concentrations. It is well known that ethanol is a chemical stress factor, which induces ROS production [39,40]. Several studies point at the activation of antioxidant and protection genes as well as glycogen and trehalose production as ethanol tolerance mechanisms [41-43]. During the first hours of fermentation, wine strains of *S. cerevisiae* increase transcription levels of stress-response genes and induce expression of proteins involved in the response to oxidative stress. Such response results in increased ROS scavenging ability of the cells, which is essential for the maintenance of their fermentative capacity. Therefore, the observed kinetics of ROS accumulation and scavenging are consistent with a transient oxidative stress during the initial phases of the fermentation process. The increase in the number of cells characterized by lower intracellular levels of ROS in Phase III might reflect the generation of subpopulations that were better adapted to unfavourable growth conditions during long-lasting batch cultures, as suggested by previous studies [37].

Notably, ROS accumulation along Phases I and II did not compromise cell viability, which remained above 90% during the three growth stages. Nevertheless, the average cell size diminished progressively along stage III, that is, during the onset of the stationary phase. While most of the cells (>90%) in stages I and II had a diameter in the range of 10–15 μm, this population fraction progressively decreased to about 50% along stage III, with a concomitant increase of the cell population in the 7–9 μm (up to about 45%) and 4–6 μm (up to about 5%). This observation is consistent with previous studies showing that cell size is negatively correlated with cell growth rate [44]. Specifically, cell size decreases as growth rate decreases and cells enter into the stationary phase. It is worth noting that the average cell size in the initial fermentation stages (10 ~ 15 μm) was significantly larger than cell sizes previously reported for other wine *S. cerevisiae* strains (ca. 4 ~ 6 μm, [45,46]). This may reflect different growth conditions (e.g. nutrient availability, ethanol concentration, oxygen availability), as cell size is linked to cell metabolism. For instance, a glucose pulse caused an increase in cell protein content (which is correlated with cell size) in carbon-limited chemostats of *S. cerevisiae*[47]. Moreover, ethanol addition also resulted in increased cell size [48]. Taken together, the larger average cell size observed at the initial fermentation stage of the batch fermentation would be the result of a combination of events, namely, the change of growth conditions when transferring cells from the inoculum culture to the bioreactor and/or the presence of ethanol virtually from the start of the cultivation. Also, the presence of a higher number of cells in the budding phase during the onset of the exponential phase that might be detected as large cells, or the presence of cell populations that might not have undergone the last cell division in the inoculum culture could be other potential explanations.

### Continuous cultures

The two first phases described on the basis of growth rate were subsequently reproduced in continuous cultures carried out at steady state conditions at dilution rates *D* = 0.27 h^−1^ defined as an approximation to μ_max_, and *D* = 0.04 h^−1^.

In these cultures, steady states were verified by on-line monitoring of the off-gas CO_2_ concentration and by measuring the concentration of the main metabolites (glucose, fructose, ethanol and glycerol) at 3, 4 and 5 residence times.

Batch phases and corresponding continuous cultures stages were compared in terms of specific growth, consumption and production specific rates (Table 1. See also Additional file 1 for complete consumption and production rates of chemostat cultures). For the calculation of these rates in batch processes, polynomial adjustments were used for the first phase (μ_max_), where exponential evolutions were observed, while linear adjustments were used for the second phase, since rates were markedly lower than during the first stage and their evolution close to linear.

**Table 1:** Comparison between reconciliated specific rates observed in batch and the equivalent chemostat cultures

		**Culture time (h)**	***Specific consumption/production rates (mmols g DCW***^***−1***^ **h**^**−1**^**)**						
			**Glucose**	**Fructose**	**Ethanol**	**Glycerol**	**Acetic acid**	**Succinic acid**	**Lactic acid**
***0.27 h***^***−1***^	***Corresponding batch phase***	8 – 17	−7.3 – -10.2	−2.2 – -7.7	6.2 – 22.0	1.08 – 3.16	0.07 – 0.19	0.04 – 0.26	0.04 – 0.08
	***Chemostat steady state***	5.5 RT (1 RT = 3.7 h)	- 14.8 ± 20	- 1.2 ± 28	17.2 ± 3.6	8.3 ± 1.1	n.d.	0.09 ± 0.01	n.d.
***0.04 h***^***−1***^	***Corresponding batch phase***	20 – 35	−4.4 – -7.2	−2.2 – -3.6	11.2 – 18.6	0.67 – 1.11	0.09 – 0.15	0.03 – 0.05	0.03 – 0.04
	***Chemostat steady state***	5.5 RT (1 RT = 25 h)	−5.0 ± 1.4	−2.3 ± 1.5	12.7 ± 1.0	0.97 ± 0.3	n.d	0.07 ± 0.01	n.d.
***0.02 h***^***−1***^	***Corresponding batch phase***	30 – 58	−2.7 – -4.8	−2.31– -3.1	7.2 – 12.8	0.4 – 0.7	0.02 – 0.1	0.02 – 0.03	0.02 – 0.06
	***Chemostat steady state***	3 RT (1 RT = 50 h)	−3.0 ± 0.6	−1.52 ± 0.6	7.9 ± 0.6	0.70 ± 0.26	0.03 ± 0.003	0.06 ± 0.01	n.d.
***0.007 h***^***−1***^	***Corresponding batch phase***	60 – 106	0 – -2.52	−1.79 – -2.07	0.78 – 8.11	0.03 – 0.36	0 – 0.04	0.02	0.003
	***Chemostat steady state***	3 RT (1 RT = 5.9 d)	−2.0 ± 0.3	−1.34 ± 0.3	5.92 ± 0.6	0.58 ± 0.35	n.d.	0.04 ± 0.004	n.d.

Media for chemostat ran at *D* = 0.04 and 0.02 h^−1^ contained 70% of the amino acid composition of the batch medium, while medium used for *D* = 0.007 h^−1^ contained 40% of the amino acid composition. For batch cultures, ranges of the specific conversion rates are given. n.d., not determined (product concentration below detection limits).

Table 1 shows that for *D* = 0.27 and 0.04 h^−1^ almost all the rates estimated for the continuous cultures are within the range of the values obtained for the mimicked batch culture. For cultures at *D* = 0.27 h^−1^, feeding medium was the same as the one used for batch cultures. However, for chemostats at *D* = 0.04 h^−1^, medium composition had to be modified for a better simulation of the conditions found in the equivalent batch phase. This was because in the batch process, the FAN was substantially consumed and $NH_{4}^{+}$ depleted during the interval considered, as observed in Figure 1. Several assays were made with best results obtained with a medium containing no $NH_{4}^{+}$ and 70% of the original amino acids content. It should be mentioned that the concentration of all amino acids was reduced in the same percentage, that is, without taking into account that the different amino acids were consumed at different rates [15]. The reason was merely practical, as it would have been cumbersome to adjust the composition of every amino acid individually. Nevertheless, results shown in Table 1 are an indication that cell metabolism of the winemaking process during the two first phases is suitably represented by the proposed series of steady states in continuous cultures. For phase I, using a dilution rate close to μ_max_ (*D* = 0.27 h^−1^) meant working at conditions with no substrates limitations, since it is known that residual substrate concentration in chemostats is only a function of the dilution rate. In particular, it increases slowly with *D* at low values but very rapidly as *D* approaches μ_max_[49,50].

Unfortunately, glucose and fructose consumption rates in stage I could not be accurately calculated. This was due to the fact that sugar concentration in both the feed tank and the bioreactor were both very high and close to each other, as sugar consumption is very low, reflecting very low cell numbers. As a result, error propagation of the glucose and fructose measures from two independent chemostat cultivations resulted in very high standard deviation values for the corresponding calculated specific consumption rates. Besides this experimental limitation, one significant divergence was found in the glycerol production rate in stage I, which was substantially higher than that in the exponential phase of the batch fermentation. Interestingly, chemostat cultures carried out under identical conditions except for a slightly lower *D* (0.25 h^−1^) and 24% glucose as single carbon source yielded glucose consumption and glycerol production rates consistent with those measured in batch fermentation [29].

For mimicking and modelling of later phases of the process, the same rationale was used as a first approach for the selection of the interval corresponding to the onset of the stationary phase. The mean growth rate calculated for this phase was μ = 0.007 h^−1^. Therefore, as a first approach, a continuous culture reproducing this phase was performed at a dilution rate *D* = 0.007 h^−1^ using the same feeding medium as for experiments at *D* = 0.04 h^−1^. Comparison between specific rates obtained at these conditions and the equivalent batch phase (III) corresponding from 35 – 100 h approximately, showed a good correspondence (Table 1). However, the chosen interval for this batch phase was very long, and thereby comprised a series of substantially different physiological states, as reflected by the wide intervals of specific conversion rates. For this reason, in a second approach, phase III was further divided into two sub-phases, an early one starting immediately after phase II with a mean specific growth rate μ = 0.02 h^−1^, followed by another (late phase III) with a mean specific growth rate μ = 0.007 h^−1^, starting at around 60 h of fermentation (Figure 1). As observed in Figure 3, amino acids were still being consumed in the early phase III, becoming limiting after 60 h of culture. Corresponding chemostats were then ran at a *D* = 0.02 h^−1^ with the same medium as for *D* = 0.04 h^−1^, while for experiments at *D* = 0.007 h^−1^, medium was redefined containing no $NH_{4}^{+}$ and 40% of the original amino acids content. This reformulation was calculated on the basis of biomass yields on nitrogen estimated from preliminary chemostats at *D* = 0.007 h^−1^ with the original medium with no $NH_{4}^{+}$ (data not shown). This further readjustment of the composition was done to improve matching with the corresponding batch conditions. Table 1 shows the comparisons between the rates obtained for these two batch sub-phases and their corresponding chemostats stages. As it can be observed, for both cases rate values are within the same ranges with one significant exception: the fructose consumption rate during the later stage, which falls below the lower bound of the corresponding specific conversion rates observed in the phase III of the batch fermentation. This observation could be related to the fact that equimolar glucose:fructose amounts are used in the chemostat feed, whereas in the onset of the stationary phase fructose concentration is higher than glucose. Also, glycerol production rate showed a tendency to be slightly higher in chemostat cultures at *D* = 0.007 h^−1^ than the corresponding values in phase III of batch fermentations. Nonetheless, such divergence was not statistically significant. As stated above, composition of individual amino acids of feed media was not modified for stage II and stage III chemostats. It is well known that nitrogen additions affect the formation of glycerol, organic acids and volatile compounds [28,51]. Therefore, it is plausible that small deviations observed in the production rates of these metabolites between batch fermentation and stage II and III chemostat cultures are the result of the standardized relative amino acid composition considered for the feed media of chemostat cultures. Indeed, nitrogen content has been proven to be a key parameter when reproducing batch wine fermentations in continuous multistage bioreactors, having a significant impact on the by-products profile [28].

### Metabolic Flux Analysis (MFA)

The changes in metabolic fluxes in the central carbon metabolism of *S. cerevisiae* over a series of steady state conditions, resembling different growth stages of wine fermentation with decreasing growth rate, were calculated using the stoichiometric model described in Additional file 2. Biomass composition (particularly the C/N ratio) was strongly affected by growth conditions, as indicated by the differences in elemental composition and major macromolecular components (protein and carbohydrates) relative abundance. Therefore, elemental and macromolecular biomass composition for each fermentation stage (Additional file 1) was incorporated in the mathematical model, allowing for a significant improvement in the adjustment of C and N balances compared to the bibliographical values. Carbon and nitrogen balances in chemostat cultures had between 2% to 11%, and 0.1 to 5% error, respectively, before the data reconciliation step. Consistency index was below 7.8 (for a redundancy of 3 and 95% significance level), indicating that no gross measurement errors of substrates and products conversion rates.

**Figure 4:**
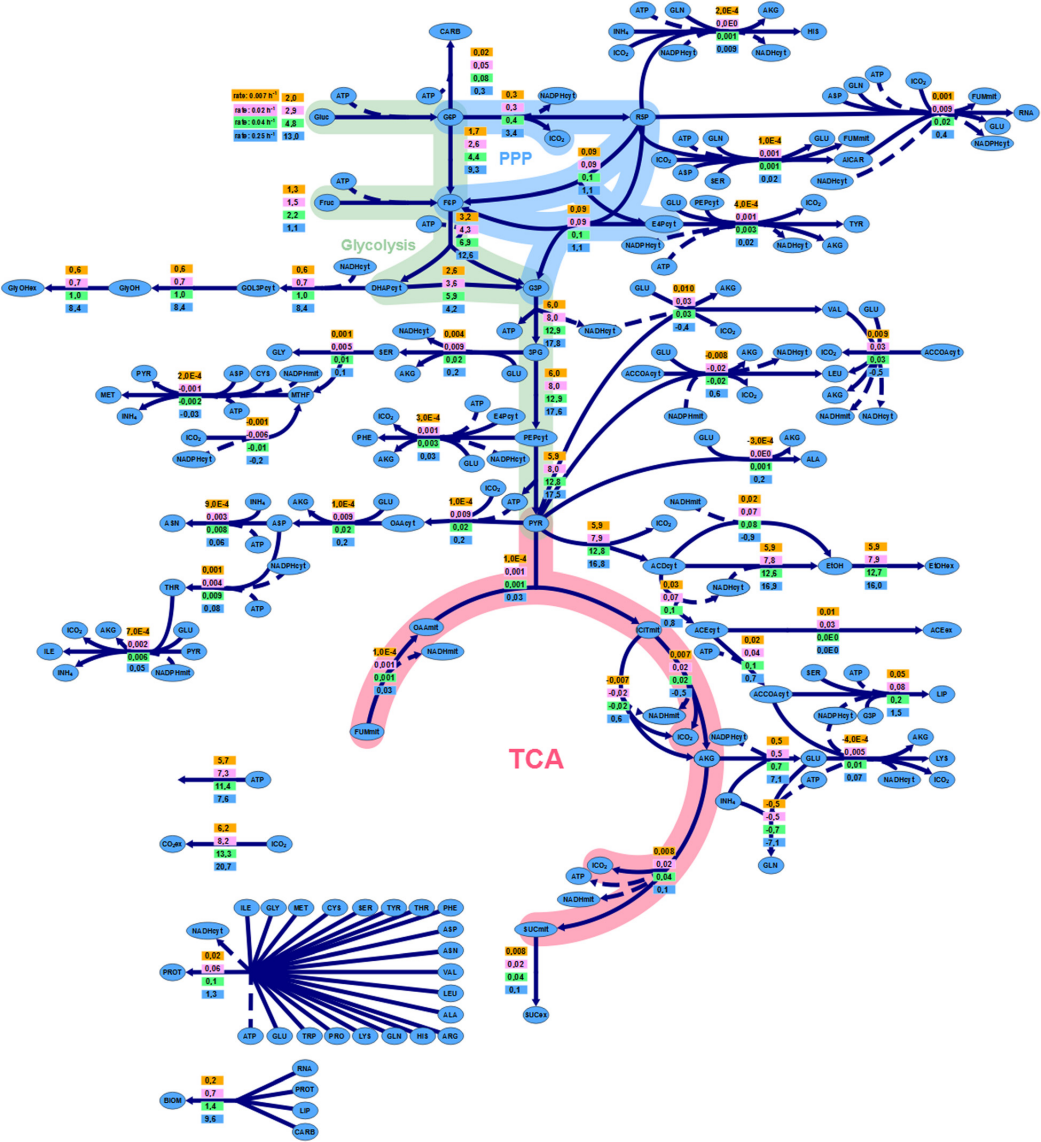
**Metabolic fluxes.** Metabolic flux distributions in the EC1118 strain during growth in chemostat cultures at different dilution rates. The values in the boxes correspond, from top to bottom, to fluxes at *D* = 0.27, 0.04, 0.02 and 0.007 h^−1^, respectively. Fluxes are given in mmol/(h · gDCW).

Overall, the metabolic flux distributions (Figure 4; see also Additional file 3 for fluxes normalised with respect to the glucose uptake flux) were coherent with previous MFA based on batch cultivations datasets [20]. As stated above, the carbon source assimilation depends on the fermentation stage: At a *D* of 0.27 h^−1^ (stage I), about 90% of the C consumption corresponds to glucose, while at the lower dilution rates of 0.04 and 0.007 h^−1^ (stages II and III) this fraction is reduced to about 70% and 60%, respectively. As expected, most of the carbon was used for energy production using the ethanol fermentative pathway; the fraction of assimilated C source used for ethanol production increased from stage I to stages II and III, from about 60% to 90%, with a concomitant decrease in the carbon source to glycerol as the *D* was reduced (from about 30% to 10%). That is, flux ratio between the ethanol and glycerol pathways increases when shifting from stage I to later stages. Notably, this effect was clearly observed despite the relative amino acid composition of the feed medium was the same for all chemostat cultures, in contrast to batch cultures where amino acids are consumed sequentially. It is well known that the nitrogen source profile influences product (i.e., ethanol, glycerol, succinate, etc.) yields [51]. Conversely, less than 1% carbon was directed to the production of other metabolites (succinate, lactate, acetate).

The calculated glycolytic and oxidative PPP branch split flux was also in agreement with previous MFA and ^13^C-MFA studies of *S. cerevisiae* under anaerobic conditions, i.e. most of carbon flux from glucose + fructose (75 ~ 95%) was channelled to glycolysis [17,20]. Specifically, the fraction of carbon directed to this pathway, the major source of NADPH required for biosynthetic pathways, diminished as the growth rate decreased.

As already described in several MFA studies [15,17,20,52], the TCA cycle operated as a two separated -oxidative and reductive- branched pathway due to the fact that in the model used the activity of Succinyl CoA synthetase was considered negligible under anaerobic conditions [13,53] and correspondingly set to zero. Also, consistent with previous studies, the anaplerotic flux was the major source of mitochondrial oxaloacetate.

## Conclusions

Our first series of results, obtained from experiments simulating the three phases of wine-growing fermentations under standard conditions suggest that steady states obtained in chemostat cultures at defined growth rates reflect the major traits of the yeast physiological state observed in the corresponding growth phase of the batch fermentation. The experimental data obtained from these cultures has been integrated into a metabolic model, allowing for the quantitative description of the metabolic state of cells (metabolic fingerprint) under each growth condition. Notably, this study has used a synthetic most medium incorporating both glucose and fructose as carbon sources, as well as potassium bisulphite to mimic cellar conditions, in contrast to previous metabolic studies based on conventional continuous and multistage continuous cultures, where glucose is often used as single carbon source, and no sulphites were added to the growth medium.

Moreover, these results proved that it is possible to define different wine-growing fermentation phases through chemostat cultures and, consequently, they have been further used to characterise metabolic changes due to temperature and increased sugar content [29], providing the basis for the future construction of a predictive physiological model aiming to mimic the global process. Conventional chemostat cultures may offer a simpler and robust alternative to continuous multistage bioreactor systems [27] for quantitative physiology studies of wine making fermentations. Nonetheless, multistage reactor systems still bring in the advantage to provide a direct means to reproduce the progressive exhaustion of nitrogen sources along the different stages, as well as reaching near-zero growth rates (as low as 0.003 h^−1^), consequently recreating both the growth and stationary phases [27]. Hence, current efforts are geared towards the establishment of chemostat cultivation strategies achieving metabolic states resembling non-proliferating cells, that is, conditions mimicking late fermentation phases with zero growth rates and increasingly higher ethanol concentration (e.g. up to 13 ~ 15%). Also, the refinement of the nitrogen sources composition of the feed media for intermediate growth stages will improve similarity of cell growth and product formation profiles of continuous cultures stages and corresponding batch phases. Furthermore, the use of ^13^C isotopic labelling techniques should allow the refinement of the metabolic flux analyses. Importantly, beyond identifying and characterising strain- and environmental-dependent physiological differences, our approach seeks to correlate physiological responses with transcriptional and metabolic changes in the future. As a first proof-of-concept of our approach, we have recently used chemostat cultures to investigate the effect of sugar concentration and temperature on exponentially growing yeast in wine fermentations [29].

The final aim is to integrate all experimental data in a framework metabolic model that can be effectively used to describe and predict a wine fermentation, as well as facilitating the rational design of reliable fermentation processes. The model could additionally be used as a tool for characterizing the metabolic behaviour of different wine yeast strains (metabolic fingerprint or phenotype), as well as for the selection of the most appropriate yeast strain as a function of the grape must composition or, in the optimal design and usage of enological additives.

## Materials and methods

### Strain

A commercial yeast strain, *Saccharomyces cerevisiae* EC1118 (Lallemand, Canada) was used in this work. This strain is considered as a model organism in the field and has been used in a wide number of physiological studies related to wine fermentations.

### Media composition

Culture medium was a modification of the MS300 medium [54]. It contained per litre: 120 g glucose; 120 g fructose; 6 g citric acid; 6 g DL-malic acid; 1.7 g YNB w/o amino acids and ammonium sulphate; 60 mg potassium bisulphite; 15 mg ergosterol; 5 mg oleic acid; 0.5 mL Tween 80; 306 mg NH_4_Cl; 29 mg L-aspartic acid; 80 mg L-glutamic acid; 52 mg L-serine; 333 mg L-glutamine; 23 mg L-histidine; 12 mg L-glycine; 13 mg L-tyrosine; 50 mg L-threonine; 245 mg L-arginine; 97 mg L-alanine; 14 mg L-cysteine; 29 mg L-valine; 21 mg L-methionine; 116 mg L-tryptophan; 25 mg L-phenylalanine; 22 mg L-isoleucine; 32 mg L-leucine; 11 mg L-lysine and 400 mg L-proline. All the amino acids required for the medium, with the exception of tyrosine, were added in a 50× solution prepared, sterilised by filtration, aliquoted, and then kept at −20°C until required. Glucose and fructose were autoclaved separately and added to the rest of medium. Concentration of glucose and fructose into the fresh medium was checked by HPLC in order to have accurate concentrations and taking possible alterations of the glucose-to-fructose ratio due to isomeration. The pH was adjusted to 3.5. Feeding media for continuous experiments had the same composition except that NH_4_Cl and the amino acids composition was adjusted to the required experiment. The same medium but with 60 g/L glucose and fructose and without anaerobic factors (ergosterol, tween 80 and oleic acid) was used for inocula growth.

### Culture conditions

All experiments were performed in 2 L bench-top bioreactors (Biostat B and Bplus, Braun Biotech, Melsungen, Germany) with a working volume of 1.5 L for batch experiments and 1 L for continuous processes. Four replicates were performed for the batch fermentation, whereas duplicate replicates were performed for each continuous cultivation condition. For inocula development, 0.1 mL cryostock of the *Saccharomyces* strain were used to inoculate 100 mL of YPD medium. The culture was grown for 48 h at 28°C and 150 rpm in a Multitron II incubator (Infors AG, Switzerland). 10 mL of this culture were then used to inoculate 100 mL of inoculum medium and incubated aerobically overnight at 28°C and 150 rpm. This culture was used to inoculate the bioreactor to an optical density (OD_600_) of 0.1. Operation parameters in the reactor were temperature 28°C, pH 3.5, and 100 rpm stirring rate. No pH control was used during batch processes, while in the continuous culture it was automatically controlled using 2 M NaOH. Data acquisition and control of the different variables was done using an in-house control software. Prior to inoculation, culture medium was sparged with 0.3 L/min of N_2_ to establish anaerobic conditions. After inoculation, N_2_ flow was diverted to the head-space to minimise the stripping of ethanol. With the same purpose, off-gas condenser was kept at 4°C throughout the process. N_2_ flow throughout the cultivation process (0.3 L/min) was controlled using a mass flow controller (Bronkhorst High Tech B.V., The Netherlands), and norprene tubing was used to avoid oxygen diffusion.Feed medium was also sparged with N_2_ throughout the experiments to maintain anaerobic conditions.

### Sampling

When required, samples were taken from the reactor in pre-chilled tubes kept on ice during processing. For elemental and macromolecular biomass composition analyses and extracellular metabolite analysis, samples were centrifuged 10 min at 10,000 rpm and 4°C. Supernatants were then filtered through 0.45 μm filters and kept at −20°C until analysis, while biomass pellets were washed twice, lyophilised and kept at −80°C until analysis. Triplicate samples were taken for all analytical measurements.

### Analytical procedures

#### Biomass dry weight

Cell biomass was monitored by measuring optical density at 600 nm (OD_600_). For cell dry weight, a known volume of culture broth was filtered through pre-weighted filters that were then washed with 2 volumes of distilled water and dried to constant weight at 105°C for 24 h.

### Sugars, organic acids, and ethanol

An Ultimate 3000 HPLC system (Dionex Corp) with an IC Sep ICE-Coregel 87H3 column (Transgenomic Inc. USA) equipped with an IR detector was used for the analysis of glucose, fructose, glycerol and ethanol while succinic, lactic, acetic and malic acids were analysed using the same equipment but with an UV detector. 15 mM sulphuric acid was used as mobile phase. Two process temperatures were used: 70°C for the analysis of fructose, and 40°C for the analyses of the other metabolites.

### CO_2_ production

Production of carbon dioxide was monitored on-line in the exhaust gas of the bioreactor using a BCP-CO_2_ sensor (BlueSens, Germany). The off-gas was passed through 2 columns containing silica gel to remove the humidity before entering the sensor.

### Yeast Assimilable Nitrogen (YAN) and Free Amino Nitrogen (FAN)

YAN was determined using 2 different commercial kits (Megazyme International, Ireland): K-NOPA which measures the PAN (primary amino acid nitrogen) and K-LARGE which measures the contribution from the side chain of L-arginine and free ammonium ions. Results from these kits were combined to determine the FAN.

### Amino acids content

Amino acids content of culture and feeding media was determined using a modification of the AccQ Tag method (Waters Corp., Milford MA, USA). Derivatisation was carried out using the AccQ Fluor reagent (6-aminoquinolyl-N-hydroxysuccinimidyl carbamate) according to the method specifications; hydrogen peroxide was added to the reaction mixture. Once derivatised, amino acids were separated and analysed using a Waters Nova-Pak C18 (4 μm, 3.9 × 150 mm) in a HPLC gradient system (Waters 600) equipped with an UV detector (Waters 2487). Detection was performed at 254 nm and α-amino-N-butyric acid (AABA) was used as internal standard.

### Flow cytometric analyses

Analyses were performed with a Guava EasyCite Mini cytometer (Millipore) with a 488-nm excitation argon-ion laser. Cell size calibration was performed using the Flow Cytometry Calibration Kit (Molecular Probes-Life Technologies) following manufacturer’s instructions. Yeast cells were harvested, washed and resuspended in PBS to a final concentration of 10^6^ cells/mL. Cell suspensions were stained according to the following procedures. Propidium iodide, PI (1 mg/mL, Sigma-Aldrich) was used to stain dead cells and dihydroethidium, DHE (12.5 μg/mL, Sigma-Aldrich) was used for ROS accumulation. Cells treated with 20% ethanol for 30 min were used as positively DHE stained cells. PI staining: 1 μL PI stock solution in PBS was added to 1 mL cell suspension just prior to the analysis and was incubated for 5 min in the dark. DHE staining: 0.2 mL DHE stock solution was added to 1 mL cell suspension and incubated at 30 °C for 30 min. Stained cells were then centrifuged and resuspended in 1 mL of PBS. After the staining step, cell samples were sonicated at low power prior to the analyses (3 pulses of 50 W, 4 s each, in a VC-50 Vibracell, Sonics & Materials) and further diluted in PBS to a concentration within the 50–500 cells/μL. 5000 cellular events were analysed for each sample (in technical triplicates). Fluorescence data for PI or DHE stained cells was collected in the channel FL2 (680 nm).

### Biomass composition analysis

#### Elemental analysis

Elemental composition of the biomass was analysed using an elemental organic analyzer Thermo EA 1108 (Thermo Scientific, Milan, Italy) following the the conditions recommended by the supplier of the instrument (helium flow at 120 mL/min, combustion furnace at 1000°C, chromatographic column oven at 60°C, and 10 mL oxygen loop at 100 kPa).

### Amino acid content

Biomass samples were first hydrolysed at 105°C for 24 h with 6 M HCl in vacuum sealed glass ampoules. After hydrolysis, samples were evaporated under vacuum, re-dissolved in 20 mM HCl, and filtered. Amino acid content of an aliquot of the filtrate is then determined using the AccQ Tag method (Waters Corp., Milford MA, USA). Derivatisation was carried out using the AccQ Fluor reagent (6-aminoquinolyl-N-hydroxysuccinimidyl carbamate) according to the method specifications. Once derivatised, amino acids were separated and analysed using a Waters Nova-Pak C18 (4 μm, 3.9 × 150 mm) in a HPLC gradient system (Waters 600) provided with an UV detector (Waters 2487). Detection was performed at 254 nm and α-amino-N-butyric acid (AABA) was used as internal standard.

### Total protein

Total protein content of the biomass was determined using the Lowry method as described in [55]. Biomass suspensions of 0.5 g/L were used for the analysis. Bovine serum albumin was used as standard.

### Total carbohydrates

Total carbohydrate content of the biomass was determined using the phenol-sulphuric method, as described in [55]. Biomass suspensions of 0.1 g/L were used for the assays. Glucose was used as standard.

### Glycogen

The glycogen content was estimated according to the method described by [56], using 20 mg of lyophilised biomass.

### Trehalose

The method described by [57] was used to estimate the trehalose content of the biomass. According to the method, 25 mg of lyophilised biomass and a standard solution of 2 g/L of trehalose were used.

### Stoichiometric model and metabolic flux analysis

The stoichiometric model used for metabolic flux analysis (Additional file 2) was adapted from the model of Varela and co-workers [20], as previously described [28]. Briefly, the described pathway network includes glycolysis, pentose phosphate pathway, the pyruvate carboxylase reaction, the synthesis of ethanol, glycerol, and acetate, the tricarboxylic acid cycle, synthesis and transport reactions for the amino acids arginine, glutamine, tryptophan, alanine, glutamate, serine, threonine, leucine, aspartate, valine, phenylalanine, and isoleucine, transport reactions for incorporation and secretion of various metabolites, and the synthesis pathways for macromolecular components. Reactions involved in either synthesis or catabolism of amino acids were included according to the following criteria: When the ratio “incorporation rate into the biomass/uptake rate” (mol gDCW^−1^ h^−1^/mol gDCW^−1^ h^−1^) of a specific amino acid was ≥ 1, the biosynthetic pathway for that amino acid was included. Conversely, when this ratio was < 1, it was assumed that there was an excess of such compound in the cell and, therefore, the corresponding degradation pathway was included. The total cell content of each amino acid residue was estimated from the molar fraction of each amino acid in the total cell protein.

Prior to metabolic flux analysis, consistency analyses for all experimental data based on elemental mass balances was performed using the methodology proposed by [58]. All experimental data passed the consistency test, considering a 95% significance level for a redundancy of 3. Metabolic fluxes were calculated using the *CellNetAnalyzer* toolbox for MATLAB developed by Klamt and co-workers [59]. Consistency index for flux analysis was always below 9.48 (redundancy of 4 at 95% confidence interval).

## Competing interests

The authors declare they have no competing interests.

## Authors’ contributions

FV, AB and PS performed the chemostat cultivations and related data analyses. AB, PS and JA carried out the data reconciliation and metabolic flux analyses. RMM, MQ and PM contributed to data interpretation and results discussion. FV and PF drafted the manuscript. RG, PM, FV, JA and PF conceived the study and its design, as well as contributing to the final drafting of the manuscript. All authors read and approved the manuscript.

## Supplementary Material

Additional file 1 **Supplementary Tables. Table S1.** Overview of the macroscopic growth parameters of the EC1118 strain growing in chemostat cultures. **Table S2.** Biomass C-molecular and macromolecular composition for *S. cerevisiae* EC1118. **Table S3.** Amino acid composition of *S. cerevisiae* EC1118. **Table S4.** Metabolic fluxes. Click here for file

Additional file 2 **Metabolic model.** Reactions in the stoichiometric model of the central carbon metabolism of *S. cerevisiae* applied in the determination of the metabolic fluxes at different dilution rates; it also includes anabolic reactions from metabolic intermediates to biosynthesis, transport reactions across the mitochondrial membrane and uptake and excretion reactions. Click here for file

Additional file 3 **Metabolic fluxes.** Metabolic flux distributions in the EC1118 strain during growth in chemostat cultures at different dilution rates. The values in the boxes correspond, from top to bottom, to fluxes at *D* = 0.27, 0.04, 0.02 and 0.007 h^−1^, respectively. Fluxes are normalized with respect glucose uptake flux (% C-mol/C-mol glucose). Click here for file
