# Supplementary material for: Use of chemostat cultures mimicking different phases of wine fermentations as a tool for quantitative physiological analysis
Source: Microb Cell Fact. 2014 Jun 13;13:85. doi: 10.1186/1475-2859-13-85 (PMC4070652; doi:10.1186/1475-2859-13-85)
Supplement: Additional file 1 — Supplementary Tables. Table S1. Overview of the macroscopic growth parameters of the EC1118 strain growing in chemostat cultures. Table S2. Biomass C-molecular and macromolecular composition for S. cerevisiae EC1118. Table S3. Amino acid composition of S. cerevisiae EC1118. Table S4. Metabolic fluxes. [file 1475-2859-13-85-S1.docx]

**Additional file 1. Supplementary Tables**

**Table S1. Overview of the macroscopic growth parameters of the EC1118 strain growing in chemostat cultures**. Reconciled measured substrates and products consumption or production rates at steady state conditions in each experiment. Substrate consumption, biomass and metabolites production rates for each strain. OUR, Oxygen Uptake Rate; CER, CO_2_ Exchange Rate; RQ Respiratory Quotient; n.d., not detectable. Consistency index h was below 7.81 for a redundancy of 3 (95 % significance level) in all cases.

|  | ***D*** (h^−1^) | | | | | | | |
| --- | --- | --- | --- | --- | --- | --- | --- | --- |
|  | 0.27 | | 0.04 | | 0.02 | | 0.007 | |
| mmol/(gDCW·h) | Bal | sd | Bal | sd | Bal | sd | Bal | sd |
| Glucose | -15 | 28 | -4.9 | 1.4 | -3.0 | 0.6 | -2.0 | 0.3 |
| Fructose | -1 | 28 | -2.3 | 1.5 | -1.5 | 0.6 | -1.3 | 0.3 |
| Glycerol | 8.1 | 1.1 | 0.97 | 0.29 | 0.69 | 0.26 | 0.58 | 0.35 |
| Etanol | 17.2 | 3.6 | 12.7 | 1.0 | 7.9 | 0.6 | 5.9 | 0.6 |
| Succinate | 0.09 | 0.01 | 0.07 | 0.01 | 0.06 | 0.01 | 0.039 | 0.004 |
| Acetate | 0.000 | 0.000 | 0.000 | 0.000 | 0.027 | 0.003 | 0.012 | 0.001 |
| Lactate | 0.000 | 0.000 | 0.000 | 0.000 | 0.000 | 0.000 | 0.000 | 0.000 |
| Asp | -0.048 | 0.009 | -0.0031 | 0.0003 | -0.0014 | 0.0001 | 0.0032 | 0.0004 |
| Glu | -0.01 | 0.03 | -0.008 | 0.001 | -0.0035 | 0.0004 | -0.0012 | 0.0001 |
| Ser | -0.042 | 0.024 | -0.007 | 0.001 | -0.0032 | 0.0003 | -0.0010 | 0.0001 |
| Gln | -0.54 | 0.09 | -0.032 | 0.003 | -0.0151 | 0.0015 | -0.0043 | 0.0004 |
| Hys | -0.030 | 0.006 | -0.0019 | 0.0002 | -0.0009 | 0.0001 | -0.00021 | 0.00003 |
| Gly | 0.014 | 0.010 | -0.0016 | 0.0002 | -0.0007 | 0.0001 | -0.00016 | 0.00004 |
| Thr | -0.054 | 0.019 | -0.006 | 0.001 | -0.0028 | 0.0003 | -0.0008 | 0.0001 |
| Arg | -0.059 | 0.067 | -0.020 | 0.002 | -0.0093 | 0.0009 | -0.0028 | 0.0003 |
| Ala | -0.006 | 0.059 | -0.014 | 0.002 | -0.0062 | 0.0007 | -0.0019 | 0.0003 |
| Tyr | -0.013 | 0.003 | -0.0009 | 0.0001 | -0.0004 | 0.00005 | -0.0001 | 0.00002 |
| Val | -0.004 | 0.013 | -0.0034 | 0.0003 | -0.0016 | 0.0002 | -0.00052 | 0.00005 |
| Met | -0.033 | 0.006 | -0.0020 | 0.0002 | -0.0009 | 0.0001 | -0.00018 | 0.00002 |
| Cys | -0.042 | 0.004 | -0.0016 | 0.0002 | -0.0008 | 0.0001 | -0.0002 | 0.0000 |
| Ile | -0.013 | 0.008 | -0.0018 | 0.0002 | -0.0008 | 0.0001 | -0.0002 | 0.0001 |
| Trp | -0.076 | 0.025 | -0.008 | 0.001 | -0.0038 | 0.0004 | -0.0007 | 0.0001 |
| Leu | -0.021 | 0.012 | -0.0034 | 0.0003 | -0.0016 | 0.0002 | -0.0005 | 0.0001 |
| Phe | -0.022 | 0.007 | -0.0020 | 0.0002 | -0.0010 | 0.0001 | -0.00026 | 0.00003 |
| Lys | -0.027 | 0.003 | 0.0034 | 0.0005 | 0.0021 | 0.0003 | 0.0010 | 0.0001 |
| NH_4_ | -0.5 | 0.2 | - | - | - | - | - | - |
| Biomass | 9.5 | 0.9 | 1.4 | 0.1 | 0.69 | 0.04 | 0.25 | 0.02 |
| CO_2_ | 19.0 | 1.8 | 13.2 | 1.0 | 8.2 | 0.6 | 6.3 | 0.5 |

**Table S2. Biomass C-molecular and macromolecular composition for *S. cerevisiae* EC1118**. Biomass C-molecular formula (A) and macromolecular formula (B) for strain EC1118 growing in chemostat cultures at different dilution rates. Elementary composition of biomass, expressed as C-molecular formula for the different growth conditions. Macromolecular components abundance is expressed as mg of component per gram of cell dry weight. C:N, carbon:nitrogen ratio; H:O, hydrogen:oxygen ratio. γ, reduction degree of the biomass. n.d., not determined.

| *D*  (h^−1^) | Elemental composition | Protein  (mg/g) | Carbohydrates  (mg/g) | Trehalose  (mg/g) | Glycogen  (mg/g) |
| --- | --- | --- | --- | --- | --- |
| 0.27 | CH_1.91_O_0.54_N_0.21_ | 473 | 161 | 95 | 11 |
| 0.04 | CH_1.80_O_0.57_N_0.14_ | 453 | 235 | 99 | 40 |
| 0.02 | CH_1.85_O_0.64_N_0.13_ | 382 | 470 | 96 | 58 |
| 0.007 | ^*^CH_1.75_O_0.56_N_0.098_ | 262 | 474 | n.d. | 121 |

* Calculated from biomass macromolecular composition.

**Table S3. Amino acid composition of *S. cerevisiae* EC1118.** Amino acid composition of the whole cell extract of EC1118 strain. Amino acid composition measured for each of the growth condition tested in chemostat cultures. Data is given as percentage of each amino acid in the total cell protein.

| **Amino acid** (mg/g _DCW_) | ***D*** (h^−1^) | | | |
| --- | --- | --- | --- | --- |
|  | **0.27** | **0.04** | **0.02** | **0.007** |
| **Asp+Asn** | 51.5 | 45.7 | 44.9 | 32.8 |
| **Ser** | 26.8 | 23.2 | 23.4 | 18.8 |
| **Glu+Gln** | 62.9 | 55.1 | 50.6 | 32.9 |
| **Gly** | 26.6 | 20.1 | 19.1 | 13.6 |
| **His** | 13.7 | 10.6 | 10 | 7.4 |
| **Arg** | 51.2 | 25.6 | 26.3 | 18.5 |
| **Thr** | 26.3 | 22.7 | 22.8 | 18.1 |
| **Ala** | 31.1 | 25.9 | 25.4 | 17.6 |
| **Pro** | 21.2 | 22.2 | 16.1 | 13.7 |
| **Cys** | n.d. | 2.1 | 1.7 | 0 |
| **Tyr** | 15.4 | 16.8 | 15.2 | 11.2 |
| **Val** | 30.9 | 25.9 | 23.1 | 16.9 |
| **Met** | n.d. | 4.8 | 0 | 0 |
| **Lys** | 45.8 | 35.2 | 34.1 | 24.8 |
| **Ileu** | 26.3 | 22.6 | 20.2 | 15.1 |
| **Leu** | 39.9 | 33.5 | 31.2 | 23.1 |
| **Phe** | 22.8 | 19.1 | 18.2 | 13.3 |
| **Total** | **492.4** | **411.1** | **382.3** | **277.8** |

**Table S4. Metabolic fluxes**. Fluxes are given in mmol/(gDCW·h)

| **Reaction** | ***D*** (h^−1^) | | | | | | | |
| --- | --- | --- | --- | --- | --- | --- | --- | --- |
|  | 0.27 | | 0.04 | | 0.02 | | 0.007 | |
|  | Bal | sd | Bal | sd | Bal | sd | Bal | sd |
| r1 | 13 | 28 | 4.8 | 1.4 | 2.93 | 0.59 | 2.00 | 0.32 |
| r2 | 1 | 28 | 2.2 | 1.5 | 1.49 | 0.61 | 1.35 | 0.34 |
| r3 | 9.3 | 0.8 | 4.36 | 0.88 | 2.61 | 0.59 | 1.70 | 0.52 |
| r4 | 13 | 1 | 6.86 | 0.38 | 4.28 | 0.29 | 3.23 | 0.32 |
| r5 | 4.2 | 1.0 | 5.89 | 0.52 | 3.59 | 0.44 | 2.63 | 0.51 |
| r6 | 17.8 | 1.2 | 12.87 | 0.71 | 7.97 | 0.54 | 5.95 | 0.58 |
| r7 | 17.6 | 1.2 | 12.85 | 0.71 | 7.96 | 0.54 | 5.95 | 0.58 |
| r8 | 17.5 | 1.2 | 12.84 | 0.71 | 7.95 | 0.54 | 5.95 | 0.58 |
| r9 | 8.36 | 0.98 | 0.97 | 0.52 | 0.69 | 0.49 | 0.60 | 0.55 |
| r10 | 8.36 | 0.98 | 0.97 | 0.52 | 0.69 | 0.49 | 0.60 | 0.55 |
| r11 | 16.8 | 1.1 | 12.8 | 0.7 | 7.9 | 0.5 | 5.9 | 0.6 |
| r12 | 15.9 | 2.3 | 12.6 | 0.8 | 7.8 | 0.6 | 5.9 | 0.6 |
| r13 | 0.1 | 2.0 | 0.1 | 0.4 | 0.1 | 0.3 | 0.0 | 0.2 |
| r14 | 0.79 | 0.15 | 0.103 | 0.035 | 0.066 | 0.051 | 0.03 | 0.22 |
| r15 | 0.72 | 0.18 | 0.101 | 0.040 | 0.038 | 0.029 | 0.021 | 0.019 |
| r16 | 3.39 | 0.69 | 0.40 | 0.28 | 0.28 | 0.26 | 0.28 | 0.36 |
| r17 | 1.13 | 0.23 | 0.13 | 0.09 | 0.09 | 0.09 | 0.09 | 0.12 |
| r18 | 1.08 | 0.24 | 0.13 | 0.09 | 0.09 | 0.09 | 0.09 | 0.12 |
| r19 | 0.033 | 0.003 | 0.001 | 0.000 | 0.001 | 0.000 | 0.000 | 0.000 |
| r20 | -0.037 | 1.057 | 0.018 | 0.197 | 0.023 | 0.145 | 0.007 | 0.073 |
| r21 | 0.069 | 1.058 | -0.017 | 0.197 | -0.022 | 0.146 | -0.007 | 0.073 |
| r22 | 0.110 | 0.093 | 0.035 | 0.052 | 0.020 | 0.040 | 0.008 | 0.023 |
| r23 | 0.033 | 0.003 | 0.001 | 0.000 | 0.001 | 0.000 | 0.000 | 0.000 |
| r24 | 0.19 | 0.21 | 0.02 | 0.04 | 0.01 | 0.03 | 0.00 | 0.03 |
| r25 | 6.62 | 1.56 | 0.69 | 0.59 | 0.52 | 0.53 | 0.54 | 0.60 |
| r26 | -6.10 | 2.00 | -0.67 | 0.62 | -0.51 | 0.55 | -0.54 | 0.61 |
| r27 | 0.19 | 0.21 | 0.02 | 0.04 | 0.01 | 0.03 | 0.00 | 0.03 |
| r28 | 0.22 | 0.20 | 0.02 | 0.04 | 0.01 | 0.02 | 0.00 | 0.01 |
| r29 | 0.15 | 0.24 | 0.00 | 0.04 | 0.00 | 0.03 | 0.00 | 0.02 |
| r30 | 0.08 | 0.17 | 0.01 | 0.03 | 0.00 | 0.02 | 0.00 | 0.02 |
| r31 | - | - | - | - | - | - | - | - |
| r32 | 0.11 | 1.00 | -0.02 | 0.19 | -0.02 | 0.14 | -0.01 | 0.07 |
| r33 | -0.03 | 0.98 | 0.03 | 0.18 | 0.03 | 0.14 | 0.01 | 0.07 |
| r34 | - | - | - | - | - | - | - | - |
| r35 | 0.05 | 0.97 | 0.03 | 0.18 | 0.03 | 0.14 | 0.01 | 0.07 |
| r36 | - | - | - | - | - | - | - | - |
| r37 | 0.03 | 0.08 | 0.00 | 0.02 | 0.00 | 0.01 | 0.00 | 0.01 |
| r38 | - | - | - | - | - | - | - | - |
| r39 | 0.05 | 0.10 | 0.01 | 0.02 | 0.00 | 0.01 | 0.00 | 0.01 |
| r40 | - | - | - | - | - | - | - | - |
| r41 | 0.074 | 0.055 | 0.012 | 0.022 | 0.005 | 0.017 | -0.0004 | 0.0099 |
| r42 | 0.062 | 0.006 | 0.008 | 0.002 | 0.0034 | 0.0011 | 0.0009 | 0.0006 |
| r43 | -0.179 | 0.127 | -0.014 | 0.020 | -0.0055 | 0.0142 | -0.0012 | 0.0078 |
| r44 | 0.149 | 0.101 | 0.012 | 0.014 | 0.0052 | 0.0102 | 0.0014 | 0.0064 |
| r45 | -0.030 | 0.077 | -0.002 | 0.014 | -0.0004 | 0.0100 | 0.0002 | 0.0045 |
| r46 | 0.017 | 0.055 | 0.003 | 0.010 | 0.0012 | 0.0071 | 0.0004 | 0.0045 |
| r47 | 0.009 | 0.077 | 0.001 | 0.014 | 0.0004 | 0.0100 | 0.0002 | 0.0055 |
| r48 | 0.046 | 0.173 | 0.007 | 0.028 | 0.003 | 0.020 | 0.0004 | 0.0084 |
| r49 | 0.034 | 0.224 | 0.012 | 0.039 | 0.0070 | 0.0269 | 0.0003 | 0.0011 |
| r50 | 0.069 | 0.100 | 0.003 | 0.020 | 0.0009 | 0.0141 | -0.0003 | 0.0065 |
| r51 | 0.5 | 0.21 | - | - | - | - | - | - |
| r52 | -0.03 | 0.06 | 0.014 | 0.002 | 0.006 | 0.001 | 0.0019 | 0.0003 |
| r53 | 0.12 | 0.07 | 0.020 | 0.002 | 0.010 | 0.001 | 0.0011 | 0.0003 |
| r54 | 0.034 | 0.009 | 0.0031 | 0.0003 | 0.0014 | 0.000145 | 0.0032 | 0.000356 |
| r55 | 0.044 | 0.004 | 0.0016 | 0.0002 | 0.0008 | 7.69E-05 | 0 | 2.21E-05 |
| r56 | 0.36 | 0.09 | 0.049 | 0.003 | 0.025 | 0.001 | 0.0072 | 0.0004 |
| r57 | -0.07 | 0.03 | 0.012 | 0.001 | 0.0061 | 0.0004 | 0.0019 | 0.0001 |
| r58 | -0.02 | 0.01 | 0.0016 | 0.0002 | 0.0007 | 0.0001 | 0.00020 | 0.00004 |
| r59 | 0.018 | 0.006 | 0.0019 | 0.0002 | 0.0009 | 0.0001 | 0.00020 | 0.00003 |
| r60 | 0.012 | 0.008 | 0.0018 | 0.0002 | 0.0008 | 0.0001 | 0.00020 | 0.00006 |
| r61 | 0.020 | 0.012 | 0.0034 | 0.0003 | 0.0016 | 0.0002 | 0.00050 | 0.00005 |
| r62 | 0.024 | 0.003 | -0.0007 | 0.0005 | -0.0001 | 0.0003 | 0.0017 | 0.0001 |
| r63 | 0.038 | 0.006 | 0.0020 | 0.0002 | 0.00090 | 0.00009 | 0.00000 | 0.00002 |
| r64 | 0.014 | 0.007 | 0.002 | 0.000214 | 0.0010 | 0.0001 | 0.00030 | 0.00003 |
| r65 | 0.022 | 0.024 | 0.007 | 0.001 | 0.0032 | 0.0003 | 0.0010 | 0.0001 |
| r66 | 0.04 | 0.02 | 0.006 | 0.001 | 0.0028 | 0.0003 | 0.0008 | 0.0001 |
| r67 | 0.06 | 0.03 | 0.008 | 0.001 | 0.0038 | 0.000377 | 0.00070 | 0.00007 |
| r68 | 0.009 | 0.003 | 0.0009 | 0.0001 | 0.00040 | 0.00005 | 0.00010 | 0.00002 |
| r69 | 0.0004 | 0.0133 | 0.0034 | 0.000349 | 0.0016 | 0.000165 | 0.0005 | 5.33E-05 |
| r70 | 0.000 | 0.000 | 0.000 | 0.000 | 0.027 | 0.003 | 0.011 | 0.001 |
| r71 | 16.0 | 3.6 | 12.7 | 1.0 | 7.9 | 0.6 | 5.9 | 0.6 |
| r72 | 8.4 | 1.1 | 1.0 | 0.3 | 0.7 | 0.3 | 0.6 | 0.4 |
| r73 | 0.110 | 0.009 | 0.035 | 0.007 | 0.020 | 0.006 | 0.008 | 0.004 |
| r74 | 20.7 | 1.8 | 13.3 | 1.0 | 8.3 | 0.6 | 6.2 | 0.5 |
| r75 | 0.0000 | 0.0000 | 0.0000 | 0.0000 | 0.0000 | 0.0000 | 0.0000 | 0.0000 |
| r76 | 0.022 | 0.002 | 0.0010 | 0.0002 | 0.00050 | 0.00014 | 0.00010 | 0.00003 |
| r77 | 6.1 | 2.7 | 11.4 | 0.8 | 7.3 | 0.6 | 5.7 | 0.6 |
| r78 | 0.39 | 0.04 | 0.017 | 0.003 | 0.009 | 0.003 | 0.0010 | 0.0006 |
| r79 | 0.27 | 0.03 | 0.083 | 0.016 | 0.049 | 0.014 | 0.019 | 0.011 |
| r80 | 1.52 | 0.15 | 0.202 | 0.040 | 0.075 | 0.022 | 0.048 | 0.027 |
| r81 | 1.27 | 0.12 | 0.149 | 0.029 | 0.064 | 0.019 | 0.018 | 0.010 |
| r82 | 9.61 | 0.88 | 1.429 | 0.079 | 0.681 | 0.041 | 0.249 | 0.017 |
