# Supplementary material for: Use of chemostat cultures mimicking different phases of wine fermentations as a tool for quantitative physiological analysis
Source: Microb Cell Fact. 2014 Jun 13;13:85. doi: 10.1186/1475-2859-13-85 (PMC4070652; doi:10.1186/1475-2859-13-85)
Supplement: Additional file 2 — Metabolic model. Reactions in the stoichiometric model of the central carbon metabolism of S. cerevisiae applied in the determination of the metabolic fluxes at different dilution rates; it also includes anabolic reactions from metabolic intermediates to biosynthesis, transport reactions across the mitochondrial membrane and uptake and excretion reactions. [file 1475-2859-13-85-S2.docx]

|  | **Glycolysis** |
| --- | --- |
| r1 | Glc + ATP ==> G6P |
| r2 | Fruc + ATP ==> F6P |
| r3 | G6P <==> F6P |
| r4 | F6P + ATP <==> DHAP + GLA3P |
| r5 | DHAP <==> GLA3P |
| r6 | GLA3P <==> 3PG + NADH + ATP |
| r7 | 3PG <==> PEP |
| r8 | PEP <==> PYR + ATP |
|  | **Glycerol metabolism** |
| r9 | DHAP + NADH <==> GL3P |
| r10 | GL3P <==> GL |
|  | **Pyruvate metabolism** |
| r11 | PYR <==> iCO_2_ + ACAL |
| r12 | NADH + ACAL <==> EtOH |
| r13 | ACAL + NADHmit <==> EtOH |
| r14 | ACAL <==> NADPH + AC |
| r15 | AC + ATP <==> ACCOA |
|  | **Pentose phosphate pathway** |
| r16 | G6P <==> 2 NADPH + iCO_2_ + R5P |
| r17 | 2 R5P <==> F6P + E4P |
| r18 | R5P + E4P <==> F6P + GAL3P |
|  | **Tricarboxylic acid cycle** |
| r19 | PYR + OAAmit <==> iCO_2_ + ICITmit + NADHmit |
| r20 | ICITmit <==> iCO_2_ + AKG + NADHmit |
| r21 | ICITmit <==> iCO_2_ + AKG + NADPHmit |
| r22 | AKG <==> iCO_2_ + SUCCmit + ATP + NADHmit |
| r23 | FUMmit <==> OAAmit + NADHmit |
|  | **Anaplerotic reaction** |
| r24 | PYR + iCO_2_ + ATP <==> OAA |
|  | **Amino acid metabolism** |
| r25 | NADPH + AKG + iNH_4_ <==> GLU |
| r26 | ATP + iNH_4_ + GLU <==> GLN |
| r27 | OAA + GLU <==> AKG + ASP |
| r28 | 3PG + GLU <==> NADH + AKG + SER |
| r29 | PYR + GLU <==> AKG + ALA |
| r30 | 2 NADPH + 2 ATP + ASP <==> THR |
| r31 | THR + NADH <==> iCO_2_ + PROPex |
| r32 | 2 PYR + ACCOA + GLU <==> 2 iCO_2_ + 2 NADH + AKG + LEU |
| r33 | ACCOA + NADHmit + GLU + VAL <==> iCO_2_ + NADH + AKG + LEU |
| r34 | AKG + LEU <==> iCO_2_ + GLU + IAMI-OH |
| r35 | 2 PYR + NADPH + GLU <==> iCO_2_ + AKG + VAL |
| r36 | AKG + VAL <==> iCO_2_ + GLU + IBUT-OH |
| r37 | 2 PEP + NADPH + E4P + ATP + GLU <==> iCO_2_ + AKG + PHE |
| r38 | AKG + PHE <==> iCO_2_ + GLU + PHEEtOH |
| r39 | PYR + NADPHmit + GLU + THR <==> iCO_2_ + AKG + iNH_4_ + ILE |
| r40 | AKG + ILE <==> iCO_2_ + GLU + AMI-OH |
| r41 | 2 NADPH + ACCOA + 3 ATP + 2 GLU <==> iCO_2_ + 2 NADH + AKG + LYS |
| r42 | 2 ATP + iNH_4_ + ASP <==> ASN |
| r43 | 2 NADPH + iCO_2_ <==> MTHF |
| r44 | SER <==> MTHF + GLY |
| r45 | 3 NADPHmit + 3 ATP + ASP + MTHF + CYS <==> PYR + iNH_4_ + MET |
| r46 | 2 PEP + NADPH + E4P + 3 ATP + GLU <==> iCO_2_ + NADH + AKG + TYR |
| r47 | NADPH + iCO_2_ + R5P + 5 ATP + iNH_4_ + GLN <==> 2 NADH + AKG + HIS |
| r48 | AKG + TRP <==> iCO_2_ + GLU + I3-EtOH |
| r49 | NADPH + AKG + ARG <==> iCO_2_ + 2 iNH_4_ + GLU + PRO |
| r50 | NADH + AC + CYS ==> 5 NADPH + ACCOA + ATP + SER |
|  | **Nitrogen uptake** |
| r51 | ATP + NH_4_ex ==> iNH_4_ |
| r52 | ATP + ALAex ==> ALA |
| r53 | ATP + ARGex ==> ARG |
| r54 | ATP + ASPex ==> ASP |
| r55 | ATP + CYSex ==> CYS |
| r56 | ATP + GLNex ==> GLN |
| r57 | ATP + GLUex ==> GLU |
| r58 | ATP + GLYex ==> GLY |
| r59 | ATP + HISex ==> HIS |
| r60 | ATP + ILEex ==> ILE |
| r61 | ATP + LEUex ==> LEU |
| r62 | ATP + LYSex ==> LYS |
| r63 | ATP + METex ==> MET |
| r64 | ATP + PHEex ==> PHE |
| r65 | ATP + SERex ==> SER |
| r66 | ATP + THRex ==> THR |
| r67 | ATP + TRPex ==> TRP |
| r68 | ATP + TYRex ==> TYR |
| r69 | ATP + VALex ==> VAL |
|  | **Product release** |
| r70 | AC ==> ACex |
| r71 | EtOH <==> EtOHex |
| r72 | GL <==> GLex |
| r73 | SUCCmit ==> SUCCex |
| r74 | iCO_2_ ==> CO_2_ex |
| r75 | DHAP ==> LACex |
|  | **Synthesis of AICAR** |
| r76 | iCO_2_ + R5P + 6 ATP + 2 GLN + ASP + SER ==> NADPH + FUMmit + 2 GLU + 9 AICAR |
|  | **Maintenance** |
| r77 | ATP ==> |
|  | **Macromolecules biosynthesis** |
|  | *Synthesis of nucleic acid* |
| r78 | 0.057 iCO_2_ + 0.048 R5P + 0.132 NADH + 0.489 ATP + 0.105 GLN + 0.075 ASP + 0.511 AICAR ==> 0.135 NADPH + 0.027 FUMmit + 0.105 GLU + NA |
|  | *Synthesis of carbohydrates* |
| r79 | G6P + ATP ==> 6 CARB |
|  | *Synthesis of lipids* |
| r80 | 0.022 GAL3P + 0.831 NADPH + 0.416 ACCOA + 0.400 ATP + 0.034 SER ==> LIP |
|  | *Synthesis of proteins* |
| r81a | 0.27 h^-1^ |
|  | 4 ATP + 0.052344 GLU + 0.052344 GLN + 0.048496 ASP + 0.066138 SER + 0.070466 ARG + 0.094046 ALA + 0.055908 THR + 0.075811 LEU + 0.067013 VAL + 0.033301 PHE + 0.049971 ILE + 0.046925 PRO + 0.076823 LYS + 0.10019 GLY + 0.003752 CYS + 0.048496 ASN + 0.006474 MET + 0.020285 TYR + 0.009742 TRP + 0.02147 HIS= 4.786 PROT |
| r81b | 0.04 h^-1^ |
|  | 4 ATP + 0.052732 GLU + 0.052732 GLN + 0.052443 ASP + 0.073552 SER + 0.0484 ARG + 0.094517 ALA + 0.061251 THR + 0.075916 LEU + 0.065404 VAL + 0.033467 PHE + 0.050062 ILE + 0.060942 PRO + 0.072938 LYS + 0.092748 GLY + 0.002989 CYS + 0.052443 ASN + 0.00345 MET + 0.025046 TYR + 0.008607 TRP + 0.02036 HIS + 3.6e-005 NADHcyt = 4.7669 PROT |
| r81c | 0.02 h^-1^ |
|  | 4 ATP + 0.053808 GLU + 0.053808 GLN + 0.054029 ASP + 0.073792 SER + 0.046254 ARG + 0.09815 ALA + 0.061934 THR + 0.075751 LEU + 0.064016 VAL + 0.033968 PHE + 0.049044 ILE + 0.045537 PRO + 0.07309 LYS + 0.091933 GLY + 0.004528 CYS + 0.054029 ASN + 0.008273 MET + 0.025584 TYR + 0.012445 TRP + 0.020026 HIS = 0.00035 NADHcyt + 4.7783 PROT |
| r81d | 0.007 h^-1^ |
|  | 4 ATP + 0.053808 GLU + 0.053808 GLN + 0.054029 ASP + 0.073792 SER + 0.046254 ARG + 0.09815 ALA + 0.061934 THR + 0.075751 LEU + 0.064016 VAL + 0.033968 PHE + 0.049044 ILE + 0.045537 PRO + 0.07309 LYS + 0.091933 GLY + 0.004528 CYS + 0.054029 ASN + 0.008273 MET + 0.025584 TYR + 0.012445 TRP + 0.020026 HIS = 0.00035 NADHcyt + 4.7783 PROT |
|  | *Synthesis of biomass* |
| r82a | 0.27 h^-1^ |
|  | 0.16798 CARB + 0.040493 RNA + 0.63291 PROT + 0.15862 LIP ==> BIOM |
| r82b | 0.04 h^-1^ |
|  | 0.3504 CARB + 0.012122 RNA + 0.49628 PROT + 0.14119 LIP ==> BIOM |
| r82c | 0.02 h^-1^ |
|  | 0.42961 CARB + 0.012487 RNA + 0.44784 PROT + 0.11007 LIP ==> BIOM |
| r82d | 0.007 h^-1^ |
|  | 0.46243 CARB + 0.004016 RNA + 0.34184 PROT + 0.19172 LIP = > BIOM |

**Abbreviations**

3PG 3-Phospho-D-glycerate

ACCOA Acetyl-CoA

ACAL Acetaldehyde

AC Acetate

ACex Extracellular Acetate

AICAR 1-(5'-Phosphoribosyl)-5-amino-4-imidazolecarboxamide

AKG 2-Oxoglutarate

ALA L-Alanine

ALAex Extracellular L-Alanine

AMI-OH Amylalcohol

ARG L-Arginine

ARGex Extracellular L-Arginine

ASN L-Asparragine

ASP L-Aspartate

ASPex Extracellular L-Aspartate

ATP Adenosin Triphosphate

BIOM Biomass

CARB Carbohydrate

CO_2_ex Extracellular CO_2_

CYS L-Cysteine

CYSex Extracellular L-Cysteine

DHAP Di-hydroxyAcetona Phosphate

E4P D-Erythrose 4-phosphate

EtOH Ethanol

EtOHex Extracellular Ethanol

Fruc D-Fructose

F6P D-Fructose 6-phosphate

FUMmit Mitochondrial Fumarate

GLA3P Glyceraldehyde 3-Phosphate

G6P D-Glucose 6-Phosphate

GLN L-Glutamine

GLNex Extracellular L-Glutamine

GLU L-Glutamate

Glc D-Glucose

GLUex Extracellular L-Glutamate

GLY Glycine

GLYex Extracellular Glycine

GL Glycerol

GLex Extracellular Glycerol

GL3P Cytosolic Glycerol 3-Phosphate

HIS L-Histidine

HISex Extracellular L-Histidine

I3-EtOH Indole-3-ethanol

IAMI-OH Isoamyl alcohol

IBUT-OH Isobutanol

ICITmit Mitochondrial isocitrate

iCO_2_ Intracellular CO_2_

ILE L-Isoleucine

ILEex Extracellular L-Isoleucine

iNH_4_ Intracellular NH_4_

LACex Extracellular Lactate

LEU L-Leucine

LEUex Extracellular L-Leucine

LIP Lipid

LYS L-Lysine

LYSex Extracellular L-Lysine

MET L-Methionine

METex Extracellular L-Methionine

MTHF 5,10-Methyltetrahydrofolate

NADH Cytosolic NADH

NADHmit Mitochondrial NADH

NADPH Cytosolic NADPH

NADPHmit Mitochondrial NADPH

NH_4_ex Extracellular NH_4_

OAA Cytosolic Oxaloacetate

OAAmit Mitochondrial Oxaloacetate

PEP Cytosolic Phosphoenolpyruvate

PHE L-Phenylalanine

PHEEtOH 2-Phenyl ethanol

PHEex Extracellular L-Phenylalanine

PRO L-Proline

PROPex Extracellular n-Propanol

PROT Protein

PYR Pyruvate

R5P D-Ribose 5-Phosphate

NA Nucleic acid

SER L-Serine

SERex Extracellular L-Serine

SUCCex Extracellular Succinate

SUCCmit Mitochondrial Succinate

THR L-Threonine

THRex Extracellular L-Threonine

TRP L-Triptophan

TRPex Extracellular L-Triptophan

TYR L_Tyrosine

TYRex Extracellular L-Tyrosine

VAL L-Valine

VALex Extracellular L-Valine
