# Supplementary figures and images for: Use of chemostat cultures mimicking different phases of wine fermentations as a tool for quantitative physiological analysis
Source: Microb Cell Fact. 2014 Jun 13;13:85. doi: 10.1186/1475-2859-13-85 (PMC4070652; doi:10.1186/1475-2859-13-85)

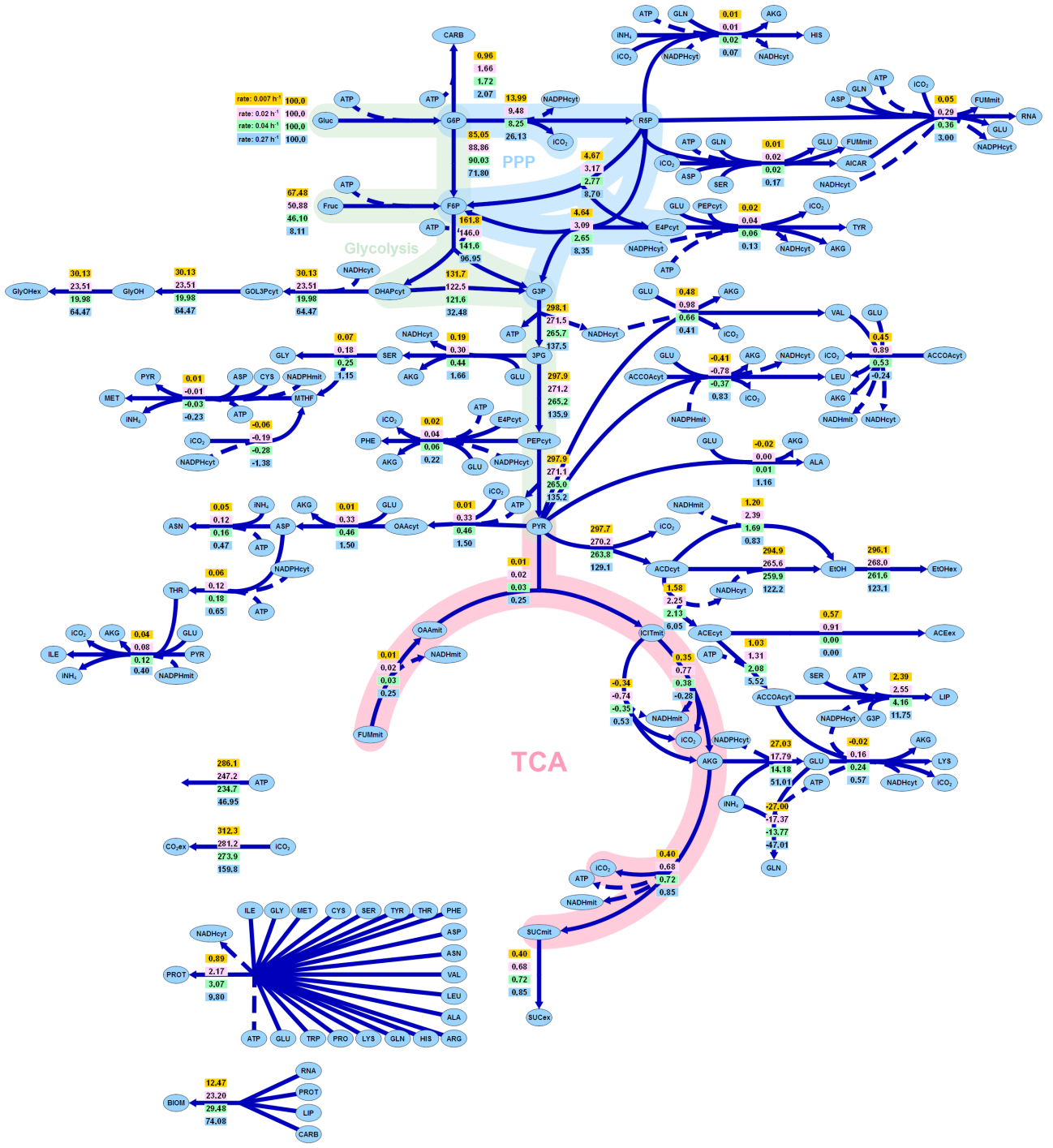

Supplement: Additional file 3 — Metabolic fluxes. Metabolic flux distributions in the EC1118 strain during growth in chemostat cultures at different dilution rates. The values in the boxes correspond, from top to bottom, to fluxes at D = 0.27, 0.04, 0.02 and 0.007 h−1, respectively. Fluxes are normalized with respect glucose uptake flux (% C-mol/C-mol glucose). [file 1475-2859-13-85-S3.docx]
